# Influence of Lowered Temperature on Efficiency of Concrete Repair with Polymer-Cement Repair Mortars

**DOI:** 10.3390/ma13194254

**Published:** 2020-09-24

**Authors:** Damian Wojnowski, Barbara Francke, Andrzej Garbacz

**Affiliations:** 1 Building Research Institute, Filtrowa 1, 00-611 Warsaw, Poland; d.wojnowski@itb.pl; 2 Department of Building Materials Engineering, Faculty of Civil Engineering, Warsaw University of Technology, Armii Ludowej 16, 00-637 Warsaw, Poland; a.garbacz@il.pw.edu.pl

**Keywords:** polymer-cement repair mortars, curing temperature, flexural and compressive strength

## Abstract

This paper presents research dealing with the evaluation of the efficiency of concrete repairs with polymer-cement mortars made at low temperatures with two types of cement and modified by copolymer acrylic-styrene. The low temperature used for the tests, of about 8 °C, is representative for Central Europe, and was established based on the analysis of mean temperatures in Poland during the last 45 years. A comparative analysis of the basic properties of the mortar tested, important from the point of view of repair efficiency, was performed, i.e., flexural and compressive strength, modulus of elasticity, adhesion to the substrate, and porosity for mortars applied and cured at 8 °C and 21°, respectively. The studies were conducted using standard methods and supported with an assessment of microscopic images (1000× magnification). It was shown that when the temperature of polymer-cement composite (PCC) mortar application is lowered to values slightly exceeding the minimum film-forming temperature (MFT) temperature of the polymer modifier, the type of cement determines the effectiveness of the repair. Only for PCC mortar with CEM I sulfate-resistant types of cement was it possible to achieve the same strength parameters as at 21 °C, during 28 days of mortar curing, and at a lowered temperature. Starting from day seven of setting at both above-mentioned temperatures, a relation between the values of the flexural and compressive strength expressed as a quotient of these values, amounting to ca. 0.14–0.19, was found.

## 1. Introduction

In Central Europe it has gradually become more common to do outdoor construction works at temperatures lower than 10 °C, due to the need to construct complex structures in a fairly short time. When the mean ambient temperature is lower than +10 °C for at least three subsequent days, special requirements apply to the execution of concrete works. The temperature of +5 °C is regarded as the boundary temperature below which the concrete mix shall be protected from heat loss [1,2,3,4,5,6]. The literature describes numerous cases of doing works in such temperature conditions, including the cases showing negative effects of such operations [7,8,9,10]. Some of the most common repair concretes and mortars are polymer-cement composites (PCC), and they are also used for repairs of concrete constructions at temperatures below 10 °C.

Polymer-cement composites are obtained by adding polymer, oligomer, or monomer to the concrete mix. After curing, the polymer and cement act as a co-binder. The amount of polymer admixtures ≤5% of the cement mass is usually insufficient for creating the separate continuous phase in the hardened concrete mix, while the polymer additives >5% of the cement mass can form such an additional, continuous network [11]. The mentioned value is not specified in the standard [12], in which polymer in polymer-cement mortar or concrete is defined as “an amount appropriate to impart specific properties”. Polymer can be added to cement mix in the form of water dispersion, redundant powder, water solution, and also as a liquid synthetic resin [11,13]. According to the chemical reactivity of the modifier, the PCC mortars can be categorized as:

- PCC–post mix: in which the polymerization runs simultaneously with the hydration of cement,
- PCC–pre mix: additives polymerized before mixing and their modifying action has a mainly physical character.

In the first type there are chemosetting synthetic resins (e.g., epoxies), or suitable monomers or pre-polymers. The second type consists of chemically inactive polymers, e.g., styrene-butadiene latex. Polymer modified cement composites have higher values of: flexural and tensile strength, adhesion, and more favorable barrier properties [11,13,14].

Table 1 presents a comparison of selected properties of polymer-cement concretes, polymer concretes (cementless), and cement concretes.

The engineering literature widely analyses the durability of polymer-cement products, e.g., by analyzing the basic relationships concerning polymer-cement materials in the relation: composition–microstructure–properties–application [15,16]. Well known and widely described in the literature is the PCC pre-mix model of setting developed by A. Beeldens et. al. [17]. The setting of the polymer-cement mixes in the case of PCC-premix consists in two processes, hydration of cement, and formation of continuous polymer film (coalescence), resulting from “consumption” of water by the cement and its partial evaporation [18]. A model is also known of forming a composite microstructure with a post-mix modifier (polymerizing after being added to the concrete mix, studied by Łukowski [19,20,21]), which revealed that the properties of polymer-cement composites greatly depend on the curing conditions. The quoted model is complementary to the model concerning composites with pre-mix modifiers. In the case of PCC-postmix, an additional chemical reaction between the resin and amine hardener takes place, leading to spatial crosslinking of the polymer. The hydration and coalescence are competitive processes. Premature formation of the polymer film hinders or even precludes the cement hydration. The kinetics of those processes should be adjusted in such way that the hydration precedes the coalescence.

The water included in the polymer dispersion is always taken into account while establishing the water-cement ratio, w/c, of the PCC mixes. It was proven that conclusions about the influence of polymer addition on the composite properties could be drawn based on the polymer content and type, which means that reasonable designing of polymer-cement composite materials is possible. As a result of the above described processes, the polymer-cement microstructure is formed with two interpenetrating nets: the polymer and the cement. The efficiency of modification of the concrete using thermoplastic polymer (PCC-premix) is dependent mainly on two polymer properties:

- minimum film-forming temperature, (MFT): a minimum temperature, above which the dispersed polymer particles can form the continuous film,
- glass transition temperature, (T_g_), i.e., the temperature of transition of the polymer from elastic state into the glass state.

Therefore, MFT should be lower than use temperature of the PCC. However, in some cases satisfactory results are also obtained with a relatively high value of MFT, e.g., for SAE (styrene—acrylic co-polymer) this temperature is 20–30 °C. This phenomenon is usually explained by a possible lowering of MFT in the alkaline environment of the cement paste [22]. Moreover, the polymers with a higher Tg usually have a higher mechanical strength [23]. The formation of the polymer-cement matrix is more complicated in the case of PCC-postmix; the formation of the polymer film is accompanied by the chemical reaction of polymer crosslinking. In practice, only epoxy resins are used here. During the last decade, research has been conducted to significantly lower the MFT temperature compared to the values given in Table 2, especially in the case of acrylic-styrene copolymers, even down to 3 °C.

Specific examples of repairs with PCC repair materials have been presented [24,25,26,27,28,29], but they are usually done without assessment of the works influence on the repair durability. Furthermore, besides the facts concerning conducting concrete works at lowered temperatures, another problem is related to sudden temperature drops during the 28-day curing period, which can result in the repair being ineffective or in the reduction of the designed life. Lower temperature during application of repair materials, especially PCC mortars can effect a repair efficiency by a decrease of the mechanical properties of mortar, and a decrease of adhesion between mortar and concrete substrate. In the first results of tests concerning the flexural and compressive strength of repairing mortars at the temperature of 8 °C, discussed in previous publications [30], it was shown at which point of the mortar curing the temperature decrease contributes most to the loss in its strength.

The aim of the study presented in this paper was to evaluate the effectiveness of polymer-cement repair mortars at temperatures occurring during repair works or mortar setting in lowered temperature, with particular emphasis on the effect of low temperatures on the properties of PCC repair mortars modified by acrylic-styrene copolymer with reduced MFT temperature up to 3 °C, and containing different cements, i.e., CEM I and CEM I sulphate resistant. A minimum temperature of 8° C was assumed in the study, after the analysis of average temperatures occurring in spring and autumn in five selected regions in Central Europe, from the period of 45 years. The basic properties of selected PCC mortars, important from the point of view of repair efficiency, i.e., flexural and compressive strength, modulus of elasticity, adhesion to the substrate, and porosity were determined. The evaluation of the effectiveness of the repair performed with polymer-cement mortars at a temperature reduced to 8 °C was carried out, taking into account the results of parallel tests on reference samples, made and cured throughout the entire setting period at a standard temperature, i.e., (21 ± 2) °C.

## 2. Materials and Methods

### 2.1. Materials

The research was carried for two repair PCC mortars containing different cements, i.e., CEM I and CEM I sulphate resistant, in both cases modified with the same acrylic-styrene copolymer with a MFT of 3 °C. The selection of repair mortars used in the tests was followed by a review of the materials produced, which confirmed that CEM I cement, with possible modifications, is mainly used for the production of repair mortars. Following the initial screening tests for different repair materials in the same group of materials, two types of PCC repair mortars, with CEM I cement and CEM I cement sulfate resistant, were selected for further research. In both tested mortars the same polymer was used, i.e., acrylic-styrene copolymer with a MFT of 3° C, but in a different amount in relation to the cement mass, i.e., p/c = 8.3% and p/c = 7.1% for Mortar I and Mortar II respectively. The characteristics of the PCC mortars were as follows:

- Mortar I–ingredients: CEM I cement (30.00%), quartz sand with a grain size of 0.1–2.0 mm (67.30%), deaerating agent (0.15%), polymer powder (2.50%), and polypropylene fibers (0.05%); mixed with water at 1 (mortar) to 0.132 (water) ratio.
- Mortar II-ingredients: CEM I sulphate resistant cement (28.00%), quartz sand with a grain size of 0.1–2.0 mm (67.15%), deaerating agent (0.15%), polymer powder (2.00), polypropylene fibers (0.15%), microsilica (2.50%), aerating agent (0.03%), and thickening agent (0.02%); mixed with water at 1 (mortar) to 0.15 (water) ratio.

The basic identification characteristics of the tested PCC mortars are shown in Table 3.

The above repair mortars should be applied with adhesion layers. In both cases, these adhesion layers were PCC mortars of the same ingredients but different in the cement type used. An adhesion layer with CEM I cement was used for Mortar I, and a adhesion layer with CEM I sulphate resistant cement for Mortar II. Other ingredients of the adhesion layers were: quartz sand with a grain size of 0.1–0.5 mm, microsilica, deaerating agent, thickening agent, polymer powder (the same as in Mortar I and II), and corrosion inhibitor.

The basic properties responsible for the repair efficiency, i.e., flexural and compressive strength, modulus of elasticity, adhesion to the substrate, and porosity were determined. A detailed analysis of increase rate of the compressive and flexural strength of mortar applied and cured at 8 °C in comparison to the increase rate of the values for products cured during the entire setting period at 21 °C was undertaken. The requirements of the mortars were checked after 28 setting days. The examinations of increase in the flexural and compressive strength were done after 1, 2, 3, 7, 14, 21, and 28 days of curing in two temperatures, i.e., 8 °C and 21 °C, and additionally after 42 days only in 8 °C. The examination after 42 days was aimed to determine if at a longer curing period the mortar made and cured at a lower temperature would reach the same strength as the value of 28 day’s strength for the product cured at 21 °C. The evaluation of the effectiveness of the repair performed with polymer-cement mortars at a temperature reduced to 8 °C was possible thanks to parallel tests on reference samples, made and cured throughout the entire setting period at standard temperature, i.e., (21 ± 2) °C.

**Figure 1:**
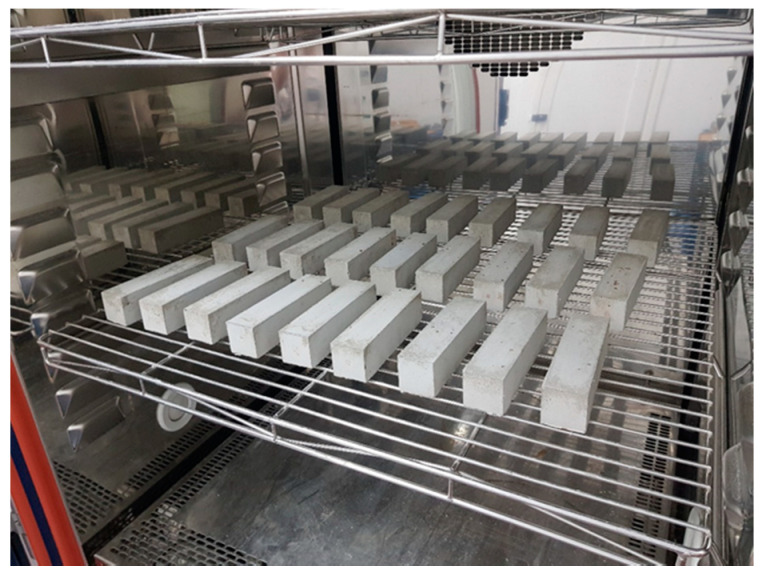
View of the samples in a climatic chamber during the test.

The test samples were prepared from both mortars at 8 °C and 21 °C. The samples were prepared in the following way:

- samples cured at (21 ± 2) °C and (60 ± 10)% RH (relative humidity), after being put in the molds and/or applied on concrete substrates were left in these conditions until reaching the required curing time. After 24 h, all samples were removed from the molds and covered with foil for the next 48 h.
- samples cured at a lowered temperature before making the mortars, the ingredients, and molds were stored at 8 °C for at least 72 h. The mortars were mixed with water in laboratory conditions, i.e., at (21 ± 2) °C and (60 ± 10)% RH, put in beam molds and/or applied on concrete substrates and immediately moved to a climatic chamber with a temperature of (8 ± 1) °C and (90 ± 10)% RH (Figure 1). After 24 h all samples were removed from the molds and covered with foil for the next 48 h.

### 2.2. Curing Conditions of PCC Mortars Samples

**Figure 2:**
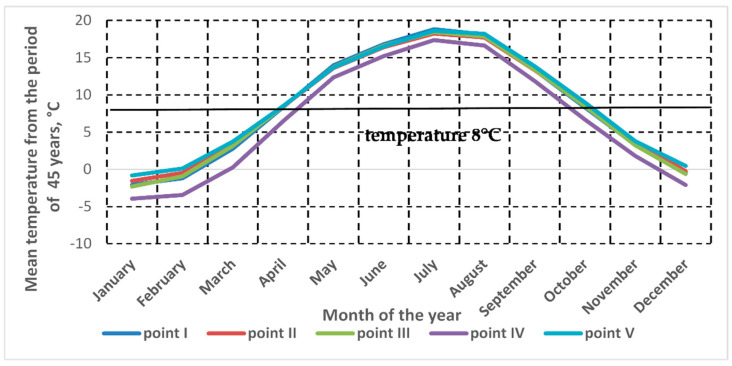
Annual distribution of average temperatures for 5 measurement points (every point from different geographic regions of Poland, i.e., one from: central, west, east, north, and south) during a period of 45-years.

Concrete constructions should be repaired with polymer-cement mortars at the recommended temperature values, ranging from 5 °C to 30 °C. These values refer both to the application and curing of polymer-cement mortars. One should remember that despite the above-mentioned performance range of the reference products, their strength characteristics are usually determined, according to standard requirements [11], in tests carried out at (21 ± 2) °C, which means the upper, optimum values of the above-mentioned operating range. Since the temperature range is extensive, before the assessment of the effectiveness of repairs done at lowered temperatures, the lowest mean temperature was established occurring in the majority of the territory of Poland in spring and autumn when repair works can already be or are still done; the obtained value was regarded as representative for the Central European area, as well as other countries worldwide. The analyses did not include the winter period because it was assumed that such works should not be carried out in winter due to the characteristics of the repair materials. From the conducted analyzes of temperature condition it was found that the average temperatures above 5 °C occur in the period from April to October, and for these months the average temperature for 5 selected measuring points in Poland (every point from different geographic regions, i.e., one from: central, west, east, north, and south) was 8 °C (Figure 2). Such a temperature value was adopted for the research assumptions. The value of relative humidity at the temperature of 8 °C was adopted in accordance with [30,31] for the stated average value of the daily course of water vapor pressure in spring and autumn, recorded in the climatic region of Central Europe at the level of 8.0 hPa to 12 hPa. This value, with reference to the maximum water vapor pressure at 8 °C, according to EN ISO 13788 [32] corresponds to a relative humidity of 90 ± 10%.

### 2.3. Methods of Tests

#### 2.3.1. Flexural and Compressive Strength Testing

The flexural and compressive strength was tested according to EN 196-1 [33], for assessing the stability of the characteristics of mortars cured at a lowered temperature. The tests were carried out for both mortars after 1, 2, 3, 7, 14, 21, and 28 days of the samples cured at the temperature and RH values of (8 ± 1) °C/(90 ± 10)%, respectively, and (21 ± 2) °C/(60 ± 10)% after 1, 2, 3, 7, 14, 21, and 28 days of curing, on six samples in each case used for flexural strength tests and twelve for compressive strength tests. The additional test after 42 days of curing was aimed to check whether the mortars cured at a lowered temperature are capable of reaching the same strength as when curing at standard temperatures, but for a longer time. The tests after 42 days on mortars cured at (8 ± 1) °C and (90 ± 10)% RH were also performed on respectively: six samples, for flexural strength tests, and twelve for compressive strength tests. The test was carried out on a CONTROLS testing strength machine with a maximum load of 3000 kN.

#### 2.3.2. Adhesion Test

The adhesion test was carried out in accordance with the EN 1542: 2000 [34] standard. The samples were made in the same way as described in point 2.1, additionally using an adhesion layer (application of the adhesion layer by intensive rubbing into a mat-damp substrate, followed by application of a repair mortar using the “wet on wet” method), and then stored for 28 days at temperatures and relative humidity of (8 ± 1) °C/(90 ± 10% and (21 ± 2) °C/(60 ± 10)%, respectively. After conditioning, 10 determinations were made for each mortar. The incisions and the sticking of the measuring stamps were made on the day preceding the tests. The test was performed with a DY-216 pull-off tester (Manufacturer-Proceq, maximum breaking force-16 kN, measuring range 0.5–16 kN).

#### 2.3.3. Modulus of Elasticity

The test of the modulus of elasticity under compression was performed according to EN 13412 [35]. For both mortars, the tests were performed after 28 days of maturing the samples at temperatures and relative humidity of (8 ± 1) °C/(90 ± 10)% and (21 ± 2) °C/(60 ± 10)%, respectively, on three samples in each case. The test was performed on a CONTROLS strength testing machine.

#### 2.3.4. Examination of Pore Distribution by Mercury Porosimetry

The pore distribution was done with the use of a mercury porosimeter by Quantachrome Instruments 1900 Corporate Drive Boyn model PM608, on cylindrical samples with a base diameter of about 2 cm. The rollers were cut from plates with dimensions of approx. 130 × 160 × 40 mm prepared as described in point 2.1 after 2 days of maturing at temperatures and relative humidity of (8 ± 1) °C/(90 ± 10)% and (21 ± 2) °C/(60 ± 10)%, respectively, followed by further maturation under the same conditions. After 28 days from forming, the samples were placed in anhydrous ethyl alcohol 99.8%, and then under reduced pressure (6 kPa) for approx. 4 h, in order to stop hydration until the test was performed. Before the pore distribution measurements were carried out, the samples were dried at 40 °C for 24 h and then stored for 4 h under reduced pressure (2 kPa) at the laboratory temperature. For each mortar, the test was carried out on 3 samples.

#### 2.3.5. Microstructure Examination

The microstructure was examined with a scanning electron microscope from JEOL 35 JSM, 1000× magnification, on cylindrical samples with the base diameter of ca. 2 cm. The cylinders were cut out from a beam prepared according to the description in item 2.1 after two days of curing the samples, and then they were divided into two groups. The first group of samples was stored for 4 h in a lowered pressure conditions (4.5 kPa), and then in lowered pressure conditions in anhydrous 99.8% ethyl alcohol. The total time of storing under reduced pressure conditions was 4 h. This was meant to stop hydration until performing the test. The other group of samples were cured for 28 days at 8 °C and 21 °C, respectively, and were then subjected to the same procedure as the samples cured for two days. Immediately before the test, the samples were removed from the alcohol and conditioned under reduced pressure conditions (2 kPa) for about 4 h. Then the samples were fractured, and the fractures were sprayed with gold for better picture contrast.

## 3. Results

This paragraph presents the results of the research which was done to determine the usability of two PCC repair mortars cured at temperature 8 °C and at the standardized laboratory temperature, i.e., (21 ± 2) °C, typically used for evaluating the performance of the reference products. The main properties influencing the efficiency of the repair mortars are given in Table 4.

**Figure 3:**
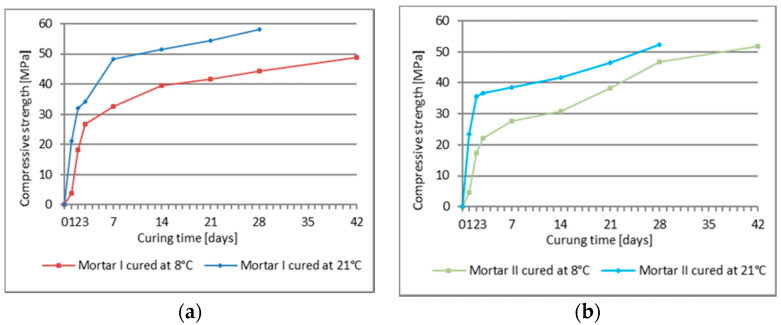
Compressive strength of mortars cured at 8 °C and 21 °C, following subsequent curing periods (average value): (**a**) PCC mortar I, (**b**) PCC mortar II.

**Figure 4:**
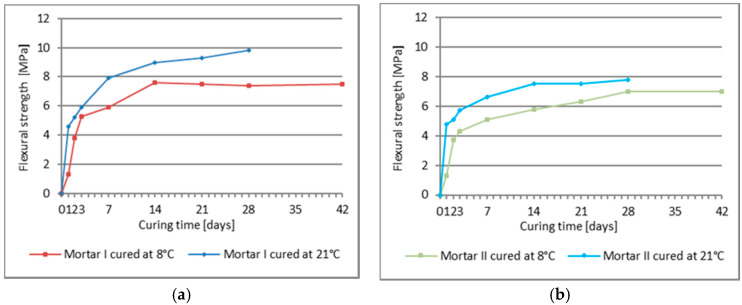
Flexural strength of mortars cured at 8 °C and 21 °C, following subsequent curing periods (average value): (**a**) mortar I, (**b**) mortar II.

The results of compressive strength tests for PCC Mortar I and PCC Mortar II cured at 8 °C and 21 °C after 1, 2, 3, 7, 14, 21, and 28 days of curing are shown in Table 5, and the results of the flexural strength for the evaluation ranges mentioned above are shown in Table 6. Moreover, Table 5 and Table 6 show the compressive and flexural strength, respectively, for mortars cured at 8 °C after 42 days. Additionally to the given average value, the tables show the coefficient of variation. Figure 3 and Figure 4 show the dynamics of changes in compressive and bending strength, respectively, for mortars curing at temperatures 8 °C and 21 °C.

**Figure 5:**
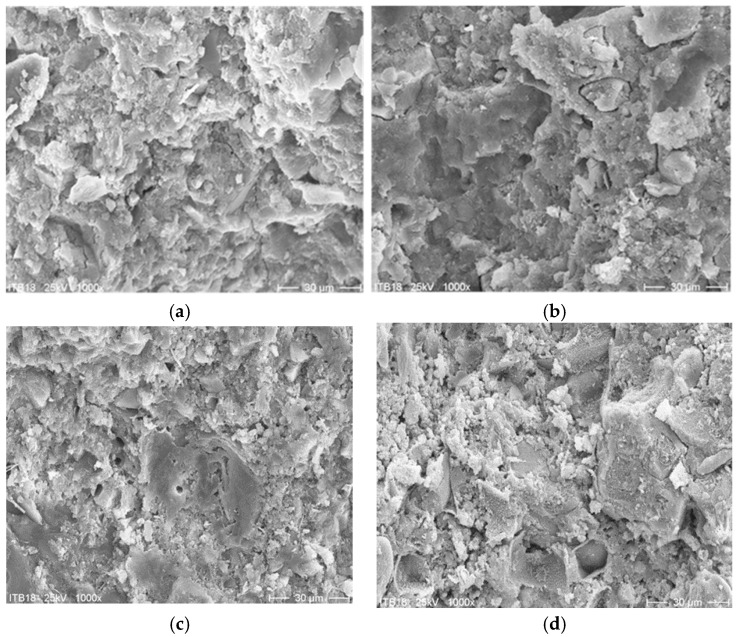
SEM image of fracture of PCC mortar I sample cured at 21 °C ((**a**)–after two days of curing, (**b**)–after 28 days of curing) and 8 °C ((**c**)–after two days of curing, (**d**)–after 28 days of curing); 1000× magnification).

**Figure 6:**
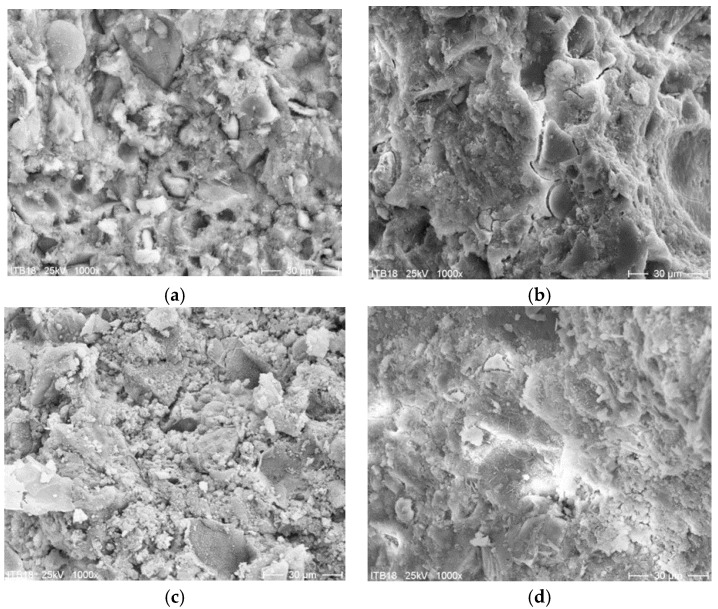
SEM image of fracture of mortar II sample cured at 21 °C ((**a**)–after two days of curing, (**b**)–after 28 days of curing) and 8 °C ((**c**)–after two days of curing, (**d**)–after 28 days of curing); 1000× magnification.

Figure 5 and Figure 6 show the microscopic images of fracture surfaces of PCC mortar I and PCC mortar II samples cured at 21 °C and 8 °C, after 2 and 28 days of setting, respectively.

## 4. Discussion

**Figure 7:**
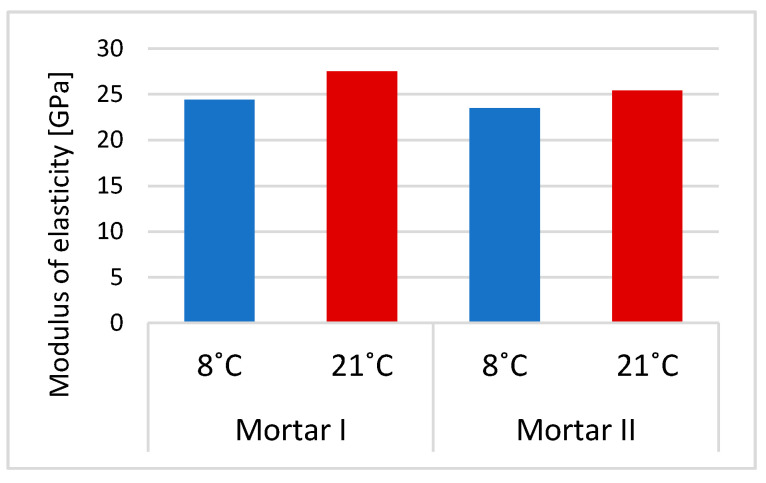
Differences in the values of the elasticity modulus for PCC mortars curing at two temperatures: 8 °C and 21 °C.

**Figure 8:**
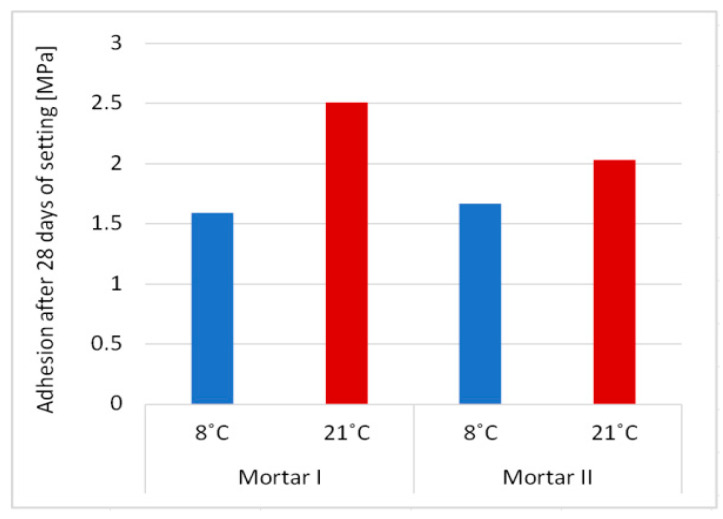
Differences in adhesion of PCC mortars curing in two temperatures: 8 °C and 21 °C.

The obtained results of the basic properties of two polymer-cement repair mortars confirm an influence of cement type on the effectiveness of the repair mortar when applied and cured at a lower temperature compared to the standard laboratory temperature, i.e., at 8 °C instead of 21 °C. The first mortar had in its composition CEM I cement, the second one had CEM I sulfate resistant cement. In both repair mortars, the same acrylic-styrene copolymer was used in a similar amount to the cement weight, i.e., in the case of mortar I–8.3%, in the case of mortar II–7.1%. Polymer used in tested mortars has the MFT temperature of 3 °C. The tests’ temperature was 8 °C which was higher than 3 °C so, according to the literature data [21], the particles of dispersed polymer at this temperature should already form a continuous polymer film. In both examined cases, the polymer film is not visible in Figure 4 and Figure 5, but this can be explained by the insufficient magnification of the microstructure images. However, in some cases satisfactory results are also obtained with relatively high value of MFT, e.g., for SAE this temperature is 20–30 °C. This phenomenon is usually explained by a possible lowering of MFT in the alkaline environment of the cement paste [13,22]. The results of total porosity [%] after 28 days of curing at temperatures of 8 °C and 21 °C for both PCC mortars indicated that the decrease of curing temperature to 8 °C causes an increase of total porosity of about only 2%. Analyzing the influence of the type of used cement on the effectiveness of repair mortars, it is clear that only CEM I sulphate-resistant cement allows obtaining similar properties for repair mortar setting at a temperature of 8 °C compared to the values obtained at 21 °C. Both the modulus of elasticity (Figure 7) and adhesion to the substrate after 28 days of setting (Figure 8) are at a similar level (modulus of elasticity: 23.5 GPa and 25.4 GPa, adhesion 1.67 MPa and 2.03 MPa). It is true that in the case of the elasticity modulus at the temperature of 8 °C, a greater variation of the results was obtained than at the temperature of 21 °C, which is visible in the higher value of the coefficient of variation, but it can be explained by more difficult temperature conditions during setting, which results in lower reproducibility of the obtained intermediate values.

In the adhesion test, there was a cohesive failure in the adhesion layer for both tested mortars. With regard to the value of adhesion for mortar I, the coefficient of variation at both temperatures is similar. Unfortunately, in the case of the repair mortar with cement CEM I, big variations were found both in the values obtained at the temperature of 8 °C and at 21 °C in the entire analyzed test range, i.e., smaller in relation to the elasticity modulus (24.4 GPa and 27.5 GPa, respectively), but significant in terms of adhesion (1.59 MPa and 2.51 MPa).

Since adhesion is one of the basic properties that determine the repair efficiency, in the case of a PCC Mortar I with cement CEM I, one may have doubts as to its effectiveness at temperatures of 8 °C, despite the fact that the MFT temperature of the applied polymer is 5 °C lower than execution and curing temperature.

**Figure 9:**
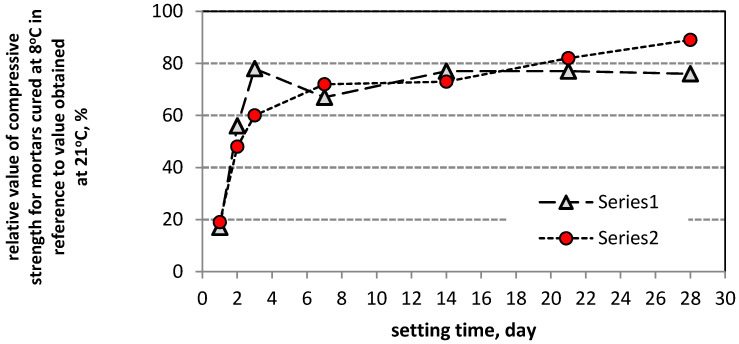
Compressive strength increase rate of mortars cured at 8 °C, in reference to the values obtained in the same period for mortar setting at 21 °C.

**Figure 10:**
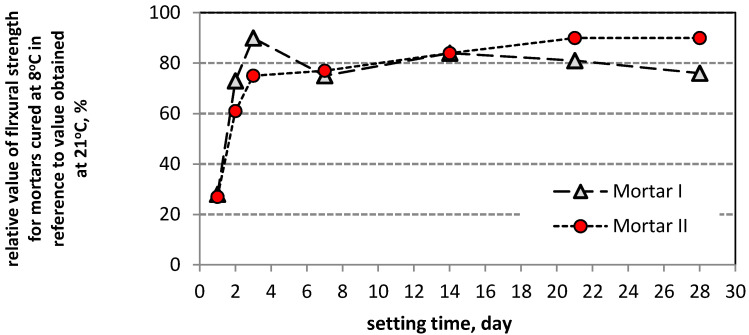
Flexural strength increase rate of mortars cured at 8 °C, in reference to the values obtained in the same period for mortar setting at 21 °C.

Similar conclusions were possible to be drawn according to the flexural and strength values obtained during the tests for the two tested repair mortars. To characterize the effect of setting time on the increase in strength of the tested mortars, the rate of growth of these two parameters over time was analyzed. For both tested PCC mortars the increase in the flexural and compressive strength was slower when they were setting at 8 °C than when the process took place at 21 °C, whereby starting from day 3 of curing, the strength increase curves are similar in all tested cases (Figure 3 and Figure 4). For the first three days, the increase in the flexural and compressive strength is much quicker when the samples are cured at 21 °C than for their setting at 8 °C. After 24 h the flexural strength of the PCC mortar cured at 21 °C is 4.6 MPa for mortar I and 4.8 MPa for mortar II, while the compressive strength amounts to 21.2 MPa and 23.4 MPa, respectively. Whereas, for the same mortars cured at 8 °C the obtained values were: flexural strength, 1.3 MPa and 1.3 MPa, and compressive strength, 3.7 MPa and 4.5 MPa. A similar disproportion is observed after 48 h when the flexural strength of the mortars cured at 21 °C amounts to 5.2 MPa and 5.1 MPa, respectively, and the flexural strength of the mortars cured at 8 °C is 3.8 MPa and 3.7 MPa, respectively; the compressive strength values amounted to 18.1 MPa and 17.2 MPa. After 72 h the values of the flexural strength for both temperatures become similar, i.e., for 21 °C, mortar I 5.9 MPa, mortar II 5.7 MPa, while for 8 °C it was 5.3 MPa and 4.3 MPa. After 72 h of setting, the discrepancies between the compressive strength values for the two test temperatures, i.e., 21 °C and 8 °C, are still significant, and amount to 34.3 MPa versus 26.7 MPa, and 35.5 MPa versus 22.0 MPa, respectively. On the following days the increase rate of the presented strength values was similar, only with minor deviations from the quoted regularity, but still at much lower values of flexural and compressive strength for the samples setting at 8 °C. It can be observed that only the PCC mortar I with cement CEM I is much more susceptible to a reduction in strength as a result of lowered temperatures, especially within the range of flexural strength whose increase is hampered after 14 days of setting. PCC mortar II at 8 °C revealed a constant increase in both flexural and compressive strength during the whole 28 days’ setting process, but unfortunately also with no significant increase in the flexural strength between setting day 28 and 42. After 42 days of curing at 8 °C, the value of the flexural strength achieved by mortar II with cement CEM I sulphate resistant was just a little bit lower than the 28 days’ strength determined in standardized laboratory conditions, i.e., at 21 °C. However, the two values mentioned are already in the same range after adjusting for the coefficient of variation of the results with respect to the mean value. After 28 days of setting at 8 °C, a constant increase in the compressive strength was observed for mortar II and the value obtained after 42 days of setting (i.e., 51.7 MPa) was only slightly lower than the 28 days’ compressive strength (i.e., 52.4 MPa) for the same product setting at 21 °C. It is clearly visible that the modification of the repair mortar, which includes CEM I sulfate resistant cement polymer with an MFT temperature of 3 °C, allows obtaining a high value of compressive and flexural strength of the repair performed, including at a temperature reduced to 8 °C. Unfortunately, in case of the repair mortar with CEM I cement, the results were not as satisfactory. Both, the flexural strength and the compressive strength were lower when the mortar was cured at the temperature of 8 °C than the corresponding values obtained at the temperature of 21 °C, although the same acrylate, styrene copolymer, was used in both repair mortars. The obtained results allow the conclusion that when the temperature of repair mortar application is lowered to values slightly exceeding the MFT temperature of the polymer additives, the type of cement determines the effectiveness of the repair mortar under such specific conditions of use. The above considerations are summarized in the diagrams presenting changes of relative compressive strength (Figure 9) and relative flexural strength (Figure 10) for mortars cured at 8 °C, in reference to the values obtained in the same period for mortars setting at 21 °C. The diagrams clearly show that in both test cases the first three days are crucial. During the first 24 h of the samples curing at 8 °C, the obtained value for flexural strength increase amounted to 27% of the strength value for the samples cured for 24 h at 21 °C, and after 28 days, 75% to 90% of the strength achieved for samples cured at 21 °C.

**Figure 11:**
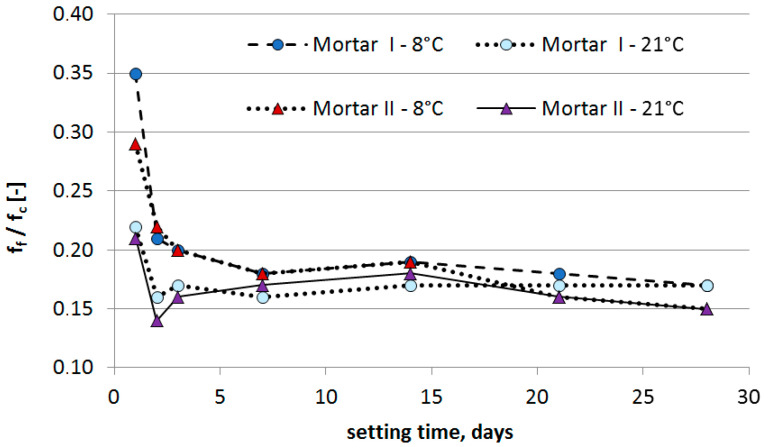
Quotient of the values of flexural and compressive strength for subsequent days of repair mortar setting at 21 °C and 8 °C, respectively.

Figure 11 presents the relation between the values of the flexural and compressive strength expressed as a coefficient f_f_/f_c_, defined as a quotient of the values mentioned above, for subsequent days of the repair mortar setting at 21 °C and 8 °C, respectively. The potential correlation was assumed to enable forecasting of the expected strength values based on the obtained partial results of the tests.

An analysis of f_f_/f_c_ quotient reveals an apparent discrepancy in the obtained values up to day 7 of the repair mortars curing both for the process temperature of 21 °C as well as for the temperature lowered to 8 °C. As curing progresses, the discrepancy is reduced. Starting from day 7, for all analyzed test results, the ratio between the flexural and compressive strength values ranges from 0.15 to 0.19, reaching 0,17 and 0,15 for Mortar I and Mortar II, respectively. A clear relationship of the achieved results at low uncertainty values leads to the conclusion that one parameter can be determined with high probability when only the other value is known.

The final stage of the research included an attempt to explain a mechanism which is the cause of lower strength values obtained for the same PCC mortars but when they are cured at 8 °C, as compared to the values obtained for mortars setting at 21 °C. Microscopic images (1000× magnification) was analyzed for mortar I and mortar II, comparing the appearance of the surface structure after 2 and 28 days, respectively, for mortars cured at two temperatures, i.e., 8 °C and 21 °C. The images shown in Figure 5 show apparent differences in the structure of mortar I at two curing temperatures. When it cures at the laboratory temperature, the CSH stage is amorphous and compact, while as curing progresses, it becomes evidently more compact. When it cures at 8 °C, the CSH stage is amorphous and quite porous, and after 28 days of curing no significant differences in the structure can be observed. The images shown in Figure 6 for mortar II show no clear differences resulting from different setting temperatures. The CSH stage is amorphous, and as setting progresses it becomes less and less porous. Such a structure of the mortar confirms the observed trends obtained in the presented research, in particular, compressive and flexural strength and adhesion to the substrate. The study of the pore distribution showed slight differences in the total porosity of both analyzed mortars, not shown clearly in the study of the image under the microscope

## 5. Conclusions

The paper presents the results of the tests for two randomly selected commercially available PCC repair mortars. Both PCC mortars were modified with the same polymer. On the basis of the results obtained the following conclusions can be drawn:

- when the temperature of PCC mortar application is lowered to values slightly exceeding the MFT temperature of the polymer additive, the type of cement mainly determines the effectiveness of the repair mortar under such specific conditions of use.
- taking into account the influence of the type of used cement on the effectiveness of repair mortars, it is clear that only CEM I sulphate-resistant cement allows obtaining similar properties for repair mortar setting at a temperature of 8 °C compared to the values obtained at 21 °C. Both the modulus of elasticity and adhesion to the substrate after 28 days of setting are at a similar level.
- for repair, mortar with CEM I sulfate resistant cement, modified by acrylate-styrene copolymer with an MFT temperature of 3 °C, allows obtaining a high value of compressive and flexural strength of the repair performed, including at a temperature reduced to 8 °C.
- in the case of the PCC repair mortar with CEM I cement, both, the flexural strength and the compressive strength were lower when the mortar was cured at the temperature of 8 °C compared to the corresponding values obtained at the temperature of 21 °C, even though the same acrylate-styrene copolymer, with an MFT temperature of 3 °C, was used.
- curing of samples at lowered temperatures of ca. 8 °C contributes to a decrease in the value of flexural and compressive strength, especially during the first days of setting. On the first day, the samples cured at 8 °C achieved a flexural strength increase amounting to 27% of the strength obtained for samples cured for 24 h at 21 °C. For the compressive strength, the value amounted to 18%. An apparent increase in this respect is observed on day 2, amounting to 60–70% for the flexural strength and 50–60% for the compressive strength. Unfortunately, the increasing trend slowed down significantly on setting day 3 at 8 °C.
- starting from day 7 of the mortar setting at both temperatures mentioned above, i.e., 8 °C and 21 °C, there was a clear relationship between the values of the flexural and compressive strength, expressed as a coefficient, and determined as a quotient of these values, amounting to 0.14–0.19.
- the microscopic image revealed the differences in the structure of mortars, resulting from different curing temperatures. The mortar cured at a lowered temperature at the first stage had a porous microstructure, while the mortar cured at a laboratory temperature had a compact microstructure. After 28 days the total porosity was higher by about 2% for both PCC mortars when they were cured at a temperature of 8 °C.

## Figures and Tables

**Table 1:** Comparison of selected properties of polymer-cement concretes (PCC), polymer concretes (PC), and Portland cement concretes [11].

Properties	Cement Concrete	PCC	PC
Density, kg/m^3^	2200–2400	1800–2200	1850–2400
Compressive strength, MPa	15–60	20–75	40–150
Flexural strength, MPa	1.1–7.2	2.5–20	4–55
Tensile strength, MPa	0.6–3.0	4–9	4–20
Modulus of elasticity, GPa	15–30	10–25	7–45

**Table 2:** Examples of values of minimum film-forming temperature (MFT) and glass transition temperature (T_g_) of the polymers [21].

Polymer	MFT, °C	T_g_, °C
SAE	20 ± 30	10 ± 20
SBR	5	10
PVAc	5	30
EVA	0 ± 10	−10 ± 15
PVA	0 ± 20	80 ± 145
PAE	Dependently on the substituent, e.g., polymethyl methacrylate (PMMA): 10	Dependently on the substituent, e.g., polymethyl methacrylate (PMMA): 105
PVP	15 ± 30	10

**Table 3:** Basic identification characteristics of the tested PCC repair mortars.

Product Characteristic	Mortar I	Mortar II
Grain size [mm]	≤2	≤2
Content of chloride ions [%]	≤0.05	≤0.05
Bulk density, g/cm^3^	1.61	1.50
Volumetric density of fresh mortar, g/cm^3^	2.18	2.08
Volumetric density of cured mortar g/cm^3^	2.20	2.18

**Table 4:** The main properties of the tested repair mortars after curing in two temperatures: 8 °C and 21 °C.

Properties	Average Value after 28 Days of Curing/Coefficient of Variation [%]			
	8 °C	21 °C	8 °C	21 °C
	Mortar I		Mortar II	
Compressive strength, MPa	44.2 (2.73)	58.1 (1.73)	46.8 (1.84)	52.4 (1.43)
Flexural strength, MPa	7.4 (0.92)	9.8 (0.93)	7.0 (1.56)	7.8 (0.77)
Adhesion strength/main failure mode, MPa	1.59 (4.32)/B *	2.51 (3.67)/B *	1.67 (4.66)/B *	2.03 (4.32)/B *
Modulus of elasticity, GPa	24.4 (1.42)	27.5 (3.74)	23.5 (4.83)	25.4 (2.46)
Total porosity, %	19.5 (1.85)	17.6 (2.15)	23.0 (1.74)	20.5 (7.93)

* B–cohesion failure in the adhesion layer.

**Table 5:** Average value of compressive strength and coefficient of variation of mortars cured at 8 °C and 21 °C, following subsequent curing periods.

Curing Time [Days]	Average Value of Compressive Strength [MPa]/Coefficient of Variation [%]			
	8 °C	21 °C	8 °C	21 °C
	Mortar I		Mortar II	
1	3.7 (2.85)	21.2 (3.95)	4.5 (6.01)	23.4 (1.19)
2	18.1 (1.99)	32.1 (2.22)	17.2 (3.38)	35.5 (1.57)
3	26.7 (1.22)	34.3 (1.32)	22.0 (2.39)	36.7 (1.06)
7	32.5 (3.94)	48.3 (1.53)	27.6 (1.94)	38.6 (1.39)
14	39.4 (2.14)	51.5 (1.76)	30.7 (3.21)	41.8 (2.36)
21	41.7 (2.93)	54.5 (1.44)	38.3 (2.99)	46.4 (1.35)
28	44.2 (2.73)	58.1 (1.73)	46.8 (1.84)	52.4 (1.43)
42	48.7 (1.79)	-	51.7 (1.17)	-

**Table 6:** Average value of flexural strength and coefficient of variation of mortars cured at 8 °C and 21 °C, following subsequent curing periods.

Curing Time [Days]	Average Value of Flexural Strength [MPa]/Coefficient of Variation [%]			
	8 °C	21 °C	8 °C	21 °C
	Mortar I		Mortar II	
1	1.3 (4.77)	4.6 (1.28)	1.3 (4.17)	4.8 (1.92)
2	3.8 (2.59)	5.2 (1.64)	3.7 (1.49)	5.1 (1.28)
3	5.3 (0.76)	5.9 (1.03)	4.3 (1.12)	5.7 (1.09)
7	5.9 (1.40)	7.9 (1.19)	5.1 (2.16)	6.6 (0.94)
14	7.6 (0.77)	9.0 (1.20)	5.8 (0.75)	7.5 (1.06)
21	7.5 (0.79)	9.3 (1.89)	6.3 (1.18)	7.5 (0.76)
28	7.4 (0.92)	9.8 (0.93)	7.0 (1.56)	7.8 (0.77)
42	7.5 (1.44)	-	7.0 (1.81)	-
